# A human patient-derived cellular model of Joubert syndrome reveals ciliary defects which can be rescued with targeted therapies

**DOI:** 10.1093/hmg/ddx347

**Published:** 2017-09-11

**Authors:** Shalabh Srivastava, Simon A Ramsbottom, Elisa Molinari, Sumaya Alkanderi, Andrew Filby, Kathryn White, Charline Henry, Sophie Saunier, Colin G Miles, John A Sayer

**Affiliations:** 1 Newcastle University, Institute of Genetic Medicine, Central Parkway, Newcastle upon Tyne, NE1 3BZ, UK; 2 Renal Services, The Newcastle Hospitals NHS Foundation Trust, Newcastle upon Tyne NE7 7DN, UK; 3 Flow Cytometry Core Facility, Faculty of Medical Sciences, Newcastle University, UK; 4 EM Research Services, Newcastle University, Newcastle upon Tyne, NE2 4HH, UK; 5 Inserm UMR-1163, Laboratory of Hereditary Kidney Diseases, 75015 Paris, France; 6 Paris Descartes Sorbonne Paris Cité University, Imagine Institute, 75015 Paris, France

## Abstract

Joubert syndrome (JBTS) is the archetypal ciliopathy caused by mutation of genes encoding ciliary proteins leading to multi-system phenotypes, including a cerebello-retinal-renal syndrome. JBTS is genetically heterogeneous, however mutations in *CEP290* are a common underlying cause. The renal manifestation of JBTS is a juvenile-onset cystic kidney disease, known as nephronophthisis, typically progressing to end-stage renal failure within the first two decades of life, thus providing a potential window for therapeutic intervention. In order to increase understanding of JBTS and its associated kidney disease and to explore potential treatments, we conducted a comprehensive analysis of primary renal epithelial cells directly isolated from patient urine (human urine-derived renal epithelial cells, hURECs). We demonstrate that hURECs from a JBTS patient with renal disease have elongated and disorganized primary cilia and that this ciliary phenotype is specifically associated with an absence of CEP290 protein. Treatment with the Sonic hedgehog (Shh) pathway agonist purmorphamine or cyclin-dependent kinase inhibition (using roscovitine and siRNA directed towards cyclin-dependent kinase 5) ameliorated the cilia phenotype. In addition, purmorphamine treatment was shown to reduce cyclin-dependent kinase 5 in patient cells, suggesting a convergence of these signalling pathways. To our knowledge, this is the most extensive analysis of primary renal epithelial cells from JBTS patients to date. It demonstrates the feasibility and power of this approach to directly assess the consequences of patient-specific mutations in a physiologically relevant context and a previously unrecognized convergence of Shh agonism and cyclin-dependent kinase inhibition as potential therapeutic targets.

## Introduction

Joubert syndrome (JBTS) is a ciliopathy syndrome which is characterized most often by cerebellar defects and retinal dystrophy. Additional phenotypes in other organ systems frequently occur, including the cystic kidney disease nephronophthisis (NPHP) (1). Positional cloning strategies and whole exome sequencing have successfully identified numerous underlying genetic causes for JBTS. *CEP290* (alias *NPHP6*) mutations underlie JBTS type 5 (2,3) and are the commonest genetic lesion associated with a cerebello-oculo-renal phenotype (4). However, clinical phenotypes associated with *CEP290* mutations may be diverse and include isolated Leber congential amaurosis (LCA) (5,6), Meckel-Gruber syndrome (7,8) and Bardet-Biedl syndrome (9).

*CEP290* encodes a 290 kDa centrosomal protein that is co-localized with gamma-tubulin at the basal body during interphase (2). During cell division CEP290 has a dynamic localization (2). Elegant work in *Chlamydomonas* has revealed that CEP290 is an integral part of the transition zone, the specialized region at the most proximal part of the cilium, which together with transition fibers forms the ciliary gate. In murine photoreceptor cells Cep290 localizes to the connecting cilium (2), a structure which represents a specialized transition zone that extends between the inner and outer segments of the photoreceptor cell (10).

We previously reported a murine model of JBTS using *LacZ* gene trapping of *Cep290*. The mice developed cystic kidney disease as well as retinal dystrophy and brain defects, recapitulating the human phenotype. Using this model, we identified abnormal Sonic hedgehog (Shh) signalling as an early feature of cystic kidney disease, preceding detectable Wnt aberration (11). Treatment with purmorphamine, which is a Hedgehog (Hh) pathway activator and facilitates the translocation of Smoothened into the primary cilia (12), partially rescued the phenotype of mutant *Cep290^LacZ/LacZ^* spheroids and is a potential, though possibly toxic therapy for ciliopathies. It is noteworthy that purmorphamine has other reported actions including the regulation of certain cell cycle control genes and promoting cellular proliferation (12,13). *CEP290* mutations have not been directly related to cell cycle defects, although a related ciliopathy gene *CEP164* (alias *NPHP15*) regulates cell cycle progression (14) and is implicated in DNA damage response signaling (15). Our earlier work reported that *Cep290* depletion, in murine kidney cells and zebrafish embryos, also causes an increase in DNA damage. This was associated with an increase in cyclin-dependent kinases 1 and 2 and prompted the treatment of murine *Cep290*-deficient cells with roscovitine, a CDK inhibitor, which rescued DNA damage and restored cellular phenotypes, including spheroid formation phenotypes (16). Pharmacological treatments for human *CEP290* ciliopathies are still lacking and therefore we aimed to explore potential drug treatments using human urine-derived renal epithelial cells (hURECs) from patients with *CEP290* mutations.

Here we characterize hURECs from a JBTS patient with *CEP290* mutations and a cerebello-retinal-renal phenotype in order to establish the ability of *ex vivo* therapies to rescue ciliary phenotypes. Cilia from this JBTS patient were abnormally long with an apparent mismatch in axonemal and ciliary membrane length. This was in comparison to hURECs derived from wild type controls and also from a patient with isolated LCA secondary to *CEP290* mutations, in whom there was no renal involvement. We used purmorphamine to rescue cilia defects, and noted a decrease in cell mitosis and cyclin-dependent kinase 5 (CDK5) levels in response to this therapy. This provided a rationale for exploring the use of siRNA directed towards CDK5 and roscovitine as a potential treatment for *CEP290* ciliopathy.

## Results

**Figure 1.**
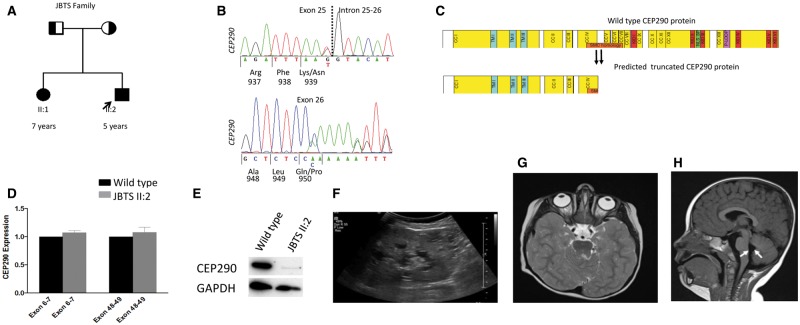
Pedigree, genetic and clinical imaging data of JBTS family H67. (**A**) Pedigree diagram showing two affected siblings (squares, males; circles females). (**B**) Sequence chromatograms showing compound heterozygous changes in *CEP290* c.2817G>T; p.K939N (predicted to affect splice donor site) and c.2848insC; p.Q950Pfs*6. (**C**) Domain structure of CEP290 protein (2479 amino acids) with predicted coiled coil domains (CC) numbered and shown in yellow; tropomyosin homology domain (TM), RepA/Rep^+^ protein KID (KID); bipartite nuclear localization signal (NLS_BP); ATP/GTP-binding site motif A (P-loop). The extent of homology with SMC proteins is indicated by an orange bar. The predicted truncation of CEP290 at amino acid positions 939 and 950 are shown. (**D**) qPCR showing relative level of CEP290 transcript measured at the 5’ (Exon 6-7) and the 3’ (Exon 48-49) ends. RNA samples from patient II: 2 hURECs show a similar level of transcript to wild type (WT, normalized to 1) indicating a lack of nonsense mediated decay (Standard error of means indicated by bars). Experiments performed in triplicate. (**E)** Western blot (cropped image) showing reduced expression of full-length wild type CEP290 protein from hURECs derived from II: 2 using a C-terminal directed CEP290 antibody. Experiments performed in triplicate. (**F)** Renal ultrasound scan of II: 2 showing cystic change within the kidney. (**G)** Axial MRI image of brain showing molar tooth sign in II: 1. (**H**) Sagittal MRI image of brain shows hypoplasia of the cerebellar vermis and enlarged mid-fourth ventricle (white arrows) in II: 1.

We studied a non-consanguineous English family in whom two children (JBTS II: 1 and JBTS II: 2) were severely affected with JBTS (Fig. 1A), manifesting as early onset nystagmus and LCA resulting in visual loss (Table 1). Both siblings have severe developmental delay, cystic kidney disease typical of NPHP (Fig. 1F) and progressive renal failure leading to end stage renal disease (ESRD) before 5 years of age. The MRI brain in JBTS II: 1 demonstrated the typical molar tooth sign (including cerebellar vermis hypoplasia and thick and abnormally oriented superior cerebellar peduncles) and enlargement of the fourth ventricle (Fig. 1G, H). Panel sequencing of ciliopathy genes allowed the identification of pathogenic compound heterozygous mutations in *CEP290* in both affected children (c.2817G>T; p.K939N and c.2848insC; p.Q950Pfs*6) leading to a predicted truncated protein, with each allele segregating from either parent (Table 1, Fig. 1A, B, C). Additional heterozygous (non-pathogenic) alleles in known ciliopathy genes were also detected including *NPHP3*: rs772079066 and *DNAH1*: rs536088715. A full list of alleles is available (Supplementary Material, Table S1, S2 and S3). qPCR of JBTS II: 2 RNA confirmed equal transcript expression at a 5’ (exon 6–7) and 3’ (exon 48–49) target but Western blotting using an antibody directed towards the C-terminus of CEP290 confirmed loss of full-length protein (Fig. 1D, E).

**Table 1.** Clinical characteristics of JBTS family (H67)

	JBTS II: 1	JBTS II: 2
Nucleotide alterations	Het c.2817G>T	Het c.2817G>T
	Het c.2848insC	Het c.2848insC
Alteration in coding sequence	Het p.K939N (splice donor site mutation)	Het p.K939N
	Het p.Q950Pfs*6 (frameshift mutation)	Het p.Q950Pfs*6
Exon (segregation)	25(M)	25(M)
	26 (P)	26 (P)
Renal USS	Increased echogenicity, corticomedullary cysts, loss of corticomedullary differentiation	Increased echogenicity, corticomedullary cysts, loss of corticomedullary differentiation
ESRD (years, months)	ESRD (1 y, 11 months)	ESRD (4 y, 9 months)
Ocular symptoms (age of onset, months)	NY (3 months)	NY (2 months)
	CA (6 months)	CA (2 months)
Central Nervous symptoms	Seizures, global developmental delay	Developmental delay
	MTS	
Other		Scoliosis

CA, congenital amaurosis (bilateral); ESRD, end-stage renal disease; Het, heterozygous in affected individual; M, Maternal mutation identified; MTS, molar tooth sign; N/A, no data available; NY, nystagmus; P, Paternal mutation identified.

**Figure 2.**
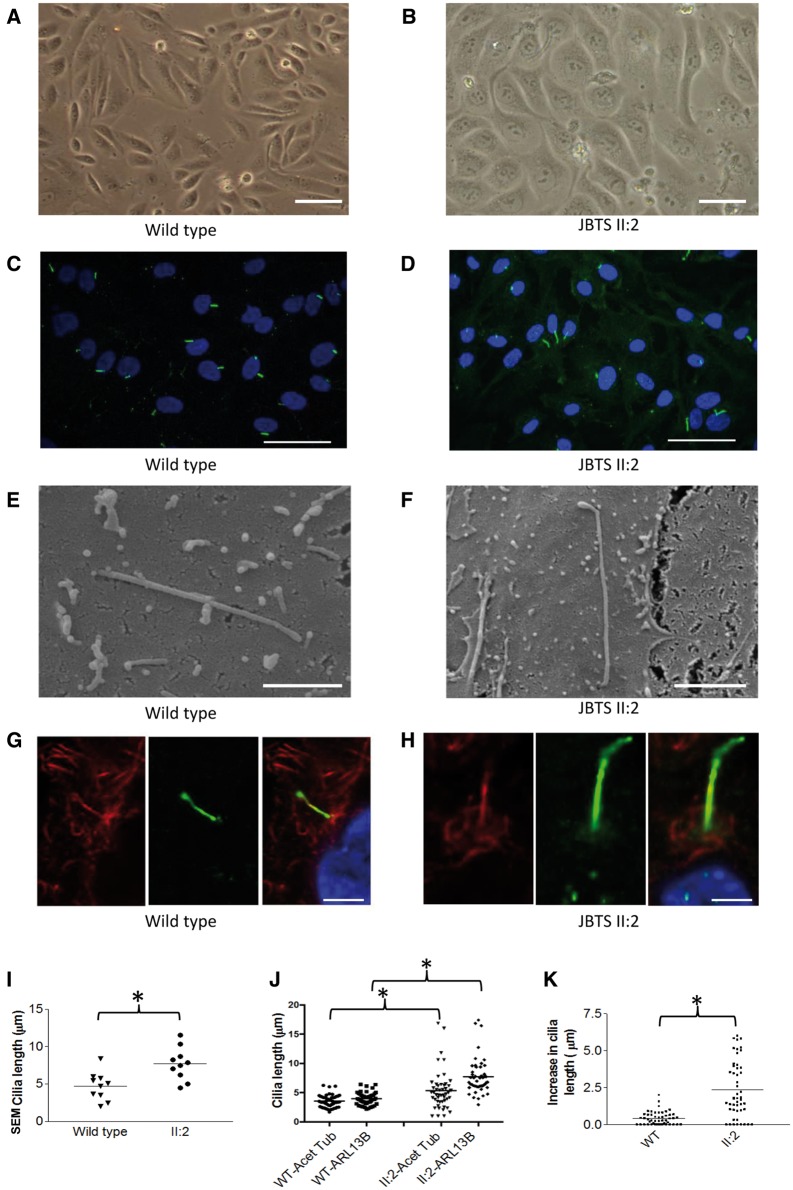
Renal epithelial cilia derived from a patient with *CEP290* mutations demonstrate length and morphological defects. (**A** and **B**) Wild type and patient JBTS II: 2 hURECs form a typical cobblestone epithelial cell layer when grown in 2D culture (Scale bar 100 µm). (**C** and **D**) Wild type and patient JBTS II: 2 hURECs following immunofluorescence imaging using anti-ARL13B (green) to identify ciliary membrane and DAPI to identify nuclei. (Scale bar 50 µm). (**E** and **F**) Scanning electron microscopy (SEM) images reveal abnormally long cilia in patient JBTS II: 2 cells with distal tip anomalies (Scale bar 2 µm). (**G** and **H**) Immunofluorescence staining using anti-alpha-acetylated tubulin (red) to identify axoneme and anti-ARL13B (green) to identify ciliary membrane in wild type and JBTS II: 2 cells (Scale bar 5um). (**I**) Dot plot with means (4.7 versus 7.7 µm) to show ciliary length measured by SEM. (n = 10 for each group, *P < 0.05, Unpaired Student’s t-test). (**J**) Dot plots with means indicated to show ciliary length measured by immunofluorescence imaging using antibodies against alpha-acetylated tubulin (Acet Tub) and ARL13B. (n = 49 for WT cilia and n = 49 for II: 2 cilia, *P < 0.0001, Unpaired Student’s *t*-test). (**K**) Dot plots with means indicated to show the increase in cilia length of each cilia (data from panel j) as determined by measuring axonemal length under immunofluorescence imaging using antibodies against alpha-acetylated tubulin (Acet Tub) and ARL13B. (*P < 0.0001, Unpaired Student’s t-test).

Prior to the onset of ESRD in patient JBTS II: 2, we were successful in culturing hURECs which were compared to wild type age matched controls (Fig. 2A, B). These primary, non-transformed renal epithelial cells provide an opportunity to carry out detailed analysis *ex vivo* of primary cells from JBTS patients to dissect the *CEP290* disease phenotype in this family. Quantification of low power immunofluorescence imaging of cells (Fig. 2C, D) revealed a significant difference in the mean ciliation rates (64% in wild-type cells versus 41% in cells from JBTS II: 2; Supplementary Material, Fig. S1a). The cilia in JBTS II: 2 were elongated (Fig. 2D) prompting more detailed imaging. Scanning electron microscopy (SEM) imaging (Fig. 2E, F) confirmed elongated primary cilia in patient JBTS II: 2 cells (mean length 7.7 µm compared to 4.9 µm in wild type) with an abnormality at the distal tip (Fig. 2F). Median lengths of cilia were 4.880 µm in wild type and 7.665 µm in JBTS II: 2 cells (Supplementary Material, Fig. S2h). Under SEM, JBTS II: 2 cilia (*n* = 10) were elongated but displayed a range of phenotypes including three that were morphologically normal, five with abnormalities of the ciliary tip (as seen in Fig. 2F) and two with an increase in their tortuosity, defined as greater than four bends or kinks in the ciliary axoneme. Cilia diameter, measured under SEM was comparable, with mean diameters of 180 nm in JBTS II: 2 versus 190 nm in wild type cilia. Length and ciliary morphology defects were confirmed by high power immunofluorescence imaging (Fig. 2G, H), using antibodies directed towards both alpha-acetylated tubulin (Acet Tub) to identify ciliary axonemes and ADP-ribosylation factor-like protein 13B (ARL13B) (17) a ciliary membrane-associated protein. The observed length difference using these two antibodies however revealed an uncoupling between the regulation of axonemal and cilia membrane length (Fig. 2I, J, K). The mean length of cilia as identified using Acet Tub increased from 3.5 µm in wild type to 5.4 µm in JBTS II: 2, but from 4.0 µm in wild type to 7.7 µm in JBTS II: 2 when identified using ARL13B antibodies to identify cilia membrane. Median cilia lengths also differed between Acet Tub and ARL13B (Supplementary Material, Fig. Sh) The mean difference in cilia length in JBTS II: 2 patients per cilium, as measured using ARL13B length minus Acet Tub length was 0.4 µm in wild type versus 2.4 µm in JBTS II: 2, with a variable rather than constant difference in length (Fig. 2K). Additional antibody staining directed towards the transition zone protein CEP162, and pericentrin, an integral component of the pericentriolar material, did not show mislocalization in JBTS II: 2 cells (Supplementary Material, Fig. S2a, b, c, d). Antibodies directed towards the intraflagellar transport protein 88 (IFT88) an axonemal protein showed increase in cilia length in JBTS II: 2 cells, with comparable cilia lengths to those measured with alpha-acetylated tubulin (Supplementary Material, Fig. S2e–h).

**Figure 3.**
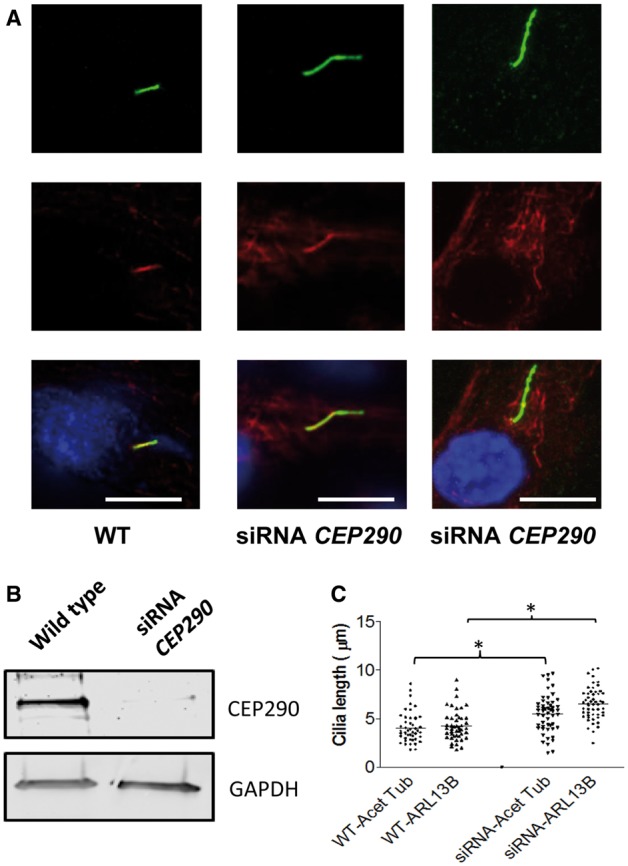
*CEP290* knockdown by siRNA in hURECs reveals a ciliary length phenotype. (**A**) Immunofluorescence imaging of cilia identified by anti-alpha-acetylated tubulin (Acet Tub red) and anti-ARL13B (ARL13B, green). Scale bar 10 μm. (**B**) Western blot (cropped image) showing loss of full length CEP290 protein in wild type hURECs following knockdown of *CEP290* using directed siRNA. (**C**). Dot plots to show ciliary length in wild type (WT) untreated and siRNA *CEP290* treated hURECs measured by immunofluorescence imaging using antibodies against alpha-acetylated tubulin (Acet Tub) and ARL13B. (n = 47 for WT cilia and n = 52 for siRNA treated cilia, *P < 0.0001, Unpaired Students t-test).

To demonstrate that these morphological defects are primarily due to a loss of function of CEP290 in the JBTS patient’s cells we sought to treat wild type hURECs with siRNA directed towards *CEP290*. siRNA treatment revealed a ciliary phenotype of increased length, resembling the patient II: 2’s cilia (Fig. 3A). *CEP290* siRNA achieved a significant knockdown of full length CEP290 protein, as determined by Western blot (Fig. 3B). We observed an increase in cilia length, as determined both by the ciliary membrane marker ARL13B and alpha acetylated tubulin (Fig. 3C).

The finding of abnormally elongated cilia in JBTS patients and following siRNA mediated knockdown of CEP290 are in contrast to the findings in another unrelated family with *CEP290* mutations. In this family there was a single affected 14 year old child with a phenotype limited to LCA, without brain or renal involvement (LCA II: 2). At this age, with documented normal renal function the development of a NPHP phenotype in this individual is extremely unlikely (18). Here the LCA phenotype was confirmed to be secondary to biallelic mutations in *CEP290* (c.297+1G>T and c.4661_4663delAAG; p.Q1554del) (Supplementary Material, Fig. S3a, b, c). Interestingly, despite biallelic mutations, CEP290 protein was evident on Western blotting of kidney protein derived from hURECs (Supplementary Material, Fig. S3d) and hURECs isolated from patient LCA II: 2 showed no significant reduction in ciliation rates or cilia length abnormality (Supplementary Material, Fig. S3e–j). These findings add some specificity to the elongated ciliary length seen in renal epithelial cells in *CEP290* patients with renal phenotypes.

**Figure 4.**
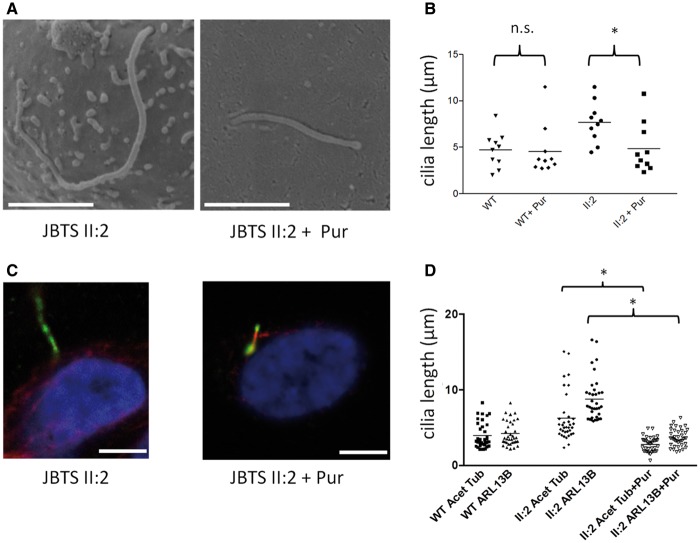
Rescue of *CEP290* mutant cilia length defects in hURECs using purmorphamine treatment. (**A**) Scanning electron microscopy (SEM) images showing cilia in patient JBTS II: 2 hURECs before and after purmorphamine (Pur) treatment. Scale bar 2 µm. (**B**) Dot plots with means showing ciliary lengths measured by SEM in wild type (WT) and patient JBTS II: 2 hURECs before and after treatment with purmorphamine (Pur). (n = 10 for each group, *P < 0.0001, Unpaired Student t-test; n.s. not significant). Untreated WT and II: 2 cilia lengths are replicated from Figure 2I. (**C**) Immunofluorescence images showing cilia in patient JBTS II: 2 hURECs before and after purmorphamine treatment. Scale bar 5 µm. (**D**) Dot plots with means to show ciliary length determined by immunofluorescence imaging in wild type (WT) patient JBTS II: 2 hURECs untreated and treated with purmorphamine using anti-alpha-acetylated tubulin (Acet Tub) and anti-ARL3B (ARL13B). (Cilia counts n = 36 for WT, n = 35 for II: 2 untreated and n = 40 for II: 2 treated; *P < 0.0001, ANOVA).

Our previous work has implicated abnormalities in the Hh pathway in *CEP290* disease mechanisms (11). Using hURECs from patient JBTS II: 2 we were able to show a rescue of both the ciliary length, with a complete rescue of increased tortuosity and bulbous phenotypes seen using SEM (*n* = 10) and restoration of the normal relationship between the axoneme identified by alpha-acetylated tubulin and ARL13B positive ciliary membrane following treatment with purmorphamine (Fig. 4A–D). Treatment with purmorphamine did not affect overall percentage ciliation rates in mutant cells (Supplementary Material, Fig. S1a). Conversely, treatment of wild-type hURECs with the Hh antagonist HPI-4 replicated the abnormally long ciliary phenotype, with increased tortuosity seen in 3 out of 10 cilia imaged using SEM. No cilia with bulbous tips were observed and there was no excess in ciliary membrane length when compared to axonemal length, using immunofluorescence microscopy, suggesting that additional factors contribute to the uncoupling of the axonal to cilia length caused by *CEP290* mutation (Supplementary Material, Fig. S4a–f).

**Figure 5.**
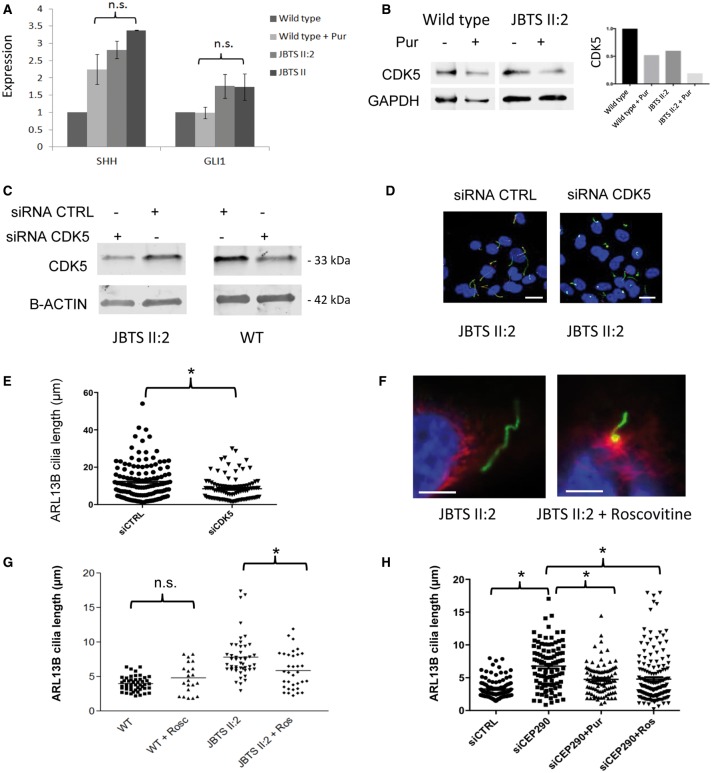
CDK5 inhibition via purmorphamine, siRNA and roscovitine rescues ciliary phenotype in CEP290 deficient cells. (**A**) Expression of *SHH* and *GLI1* RNA transcripts in hURECs from wild type controls and JBTS II: 2 before and after treatment with purmorphamine (Pur). Expression was normalized to control genes GAPDH and HPRT. Error bars indicate standard deviation; n.s., not significant, ANOVA. Experiments performed in triplicate. (**B**) Western blot (cropped image) of CDK5 levels following treatment with purmorphamine in wild type and JBTS II: 2 hURECs. Quantification (right) normalized to GAPDH expression. Experiments were performed in triplicate. (**C**) Western blot (cropped image) of siRNA CDK5 and control (CTRL) siRNA treatment of JBTS II: 2 and wild type (WT) hURECs shows a reduction in CDK5 protein expression. Beta-actin (B-ACTIN) loading control. Experiments performed in triplicate. (**D**) Low power immunofluorescence images (alpha-acetylated tubulin, red; ARL13B green, DAPI, blue) of hURECs from JBTS II: 2 after treatment with control siRNA (siCTRL) or siRNA CDK5 (siCDK5) (scale bar 10 µm). (**E**) Dot plot with means to show quantification of cilia length of hURECs from JBTS II: 2 (shown in d) after treatment with control siRNA (siCTRL) or siRNA CDK5 (siCDK5) (*P < 0.001, unpaired Student's *t*-test, n = 129 siCTRL, n = 95 siCDK5, median values siCTRL 10.43 µm, siCDK5 7.356 µm). (**F**) High power immunofluorescence images (alpha-acetylated tubulin, red; ARL13B green, DAPI, blue) of hURECs from JBTS II: 2 before and after treatment with roscovitine. Scale bar 5 µm. (**G**) Dot plot with means to show quantification of cilia length using ARL13B in wild type (WT, n = 48), wild type treated with roscovitine (WT + Rosc, n = 21), JBTS II: 2 hURECs (n = 48) and JBTS II: 2 treated with roscovitine (Ros, n = 34) (n.s. not significant, *P = 0.004, unpaired Student's *t*-test). (**H**) Dot plot with means to show quantification of cilia length using ARL13B in control siRNA (siCTRL, n = 109), siRNA CEP290 (siCEP290, n = 100), siRNA CEP290 + purmorphamine (siCEP290+Pur, n = 95) and siRNA Cep290 + roscovitine (siCEP290 + Ros, n = 162) treated hURECs (*P < 0.0001, ANOVA).

It is known that Hh agonists such as purmorphamine affect different molecular pathways, in particular proliferation, albeit in a cell type specific manner. For example, purmorphamine may in some cases promote proliferation (19) and in others suppress proliferation (20). The study of murine *Cep290* mutant cell lines has suggested that CDK inhibition may have positive effects in JBTS cellular phenotypes (16). In addition, murine models of polycystic kidney disease (21) suggest that CDK inhibition may be therapeutic option for treating cystic kidney disease. hURECs derived from JBTS II: 2 showed a small increase in SHH expression as determined by quantitative PCR in response to treatment with purmorphamine (Fig. 5A) which was not significantly different from the response to purmorphamine in control hURECs. There was a lack of response in GLI1, a Hh pathway effector, following purmorphamine treatment, which was not significantly different in both wild type and JBTS II: 2 hURECs (Fig. 5A). During renal development (22) and, as was shown recently, during renal fibrosis, SHH signalling acts in a paracrine fashion. In adult murine kidney tissue, expression of Shh was limited to the tubular epithelium of the papillary collecting duct whilst its effector Gli1 was expressed in interstitial cells only (23). Therefore hUREC cells alone may lack the ability to mediate increases in GLI1. The lack of GLI1 response in hUREC cells also raises the possibility that purmorphamine may be acting in an alternate pathway to rescue the cilia phenotype. We noted that treatment with purmorphamine reduced the percentage of cycling cells in mutant hURECs (Supplementary Material, Fig. S1b) and that CDK5 protein levels were reduced in both wild type and JBTS II: 2 cells following purmorphamine treatment (Fig. 5B). This suggests either a convergence of Hh and CDK pathways or that purmorphamine rescue was mediated by CDK inhibition. To investigate a role for CDK5 in cilia length regulation we show that siRNA directed towards CDK5 is able to rescue the increased cilia length phenotype in hURECs from JBTS II: 2 (Fig. 5C, D, E). This provided a rationale for using direct CDK inhibition in *CEP290* mutant hURECs. Mutant cilia from JBTS II: 2 responded to treatment with the CDK inhibitor roscovitine showing a significant reduction in ciliary length, as measured by ARL13B (Fig. 5F, G) whilst overall percentage ciliation rates were unchanged (Supplementary Material, Fig. S1a). When ciliary axonemes were measured with both alpha-acetylated tubulin and ARL13B we show that despite the reduction in cilia length the mismatch in measured length remained evident (Supplementary Material, Fig. S5a, b). The response to both purmorphamine and roscovitine treatment was also confirmed in wild type hURECs treated with siRNA directed towards *CEP290*. The abnormally long cilia, measured by ARL13B, produced by siRNA *CEP290* knockdown cells was rescued by both purmorphamine and roscovitine treatment (Fig. 5H) whilst overall ciliation rates remained unchanged (Supplementary Material, Fig. S5c, d).

## Discussion

The clinical phenotype of *CEP290* mutations, as with that of other JBTS and related ciliopathy genes, consists of a broad spectrum (24). However, data are lacking concerning detailed cellular, and in particular cilia phenotypes of JBTS mutations in patients. By studying primary, non-transformed renal epithelial cells derived from a JBTS patient with a cerebello-retinal-renal phenotype carrying pathogenic compound heterozygous *CEP290* mutations, we observed abnormally long cilia with a mismatch in cilia staining between alpha-acetylated tubulin, an axonemal protein, and ARL13B, a ciliary membrane associated protein. An increased ciliary length phenotype in hURECs was not seen in a patient with LCA limited *CEP290* associated disease, suggesting tissue specific cilia defects may be seen *in vivo*.

It has been previously demonstrated that CEP290 protein located within the transition zone can bridge between the cilia membrane and the axoneme as it directly binds to cellular membranes through an N-terminal domain and to microtubules through a domain located within its myosin-tail homology domain (25). Loss of a functional CEP290 protein, as in JBTS II: 2 patient’s hURECs, might therefore directly lead to an uncoupling of cilia membrane and axoneme. Importantly we could recapitulate this elongated cilia phenotype by using siRNA mediated knockdown of CEP290 in wild type hURECs, thus unequivocally implicating CEP290 in this process. To our knowledge, this is the first time that loss of function of CEP290 has been shown to lead to an elongation of cilia, rather than an impairment of cilia formation which has been shown in murine (26) and cellular models (27).

Control of primary ciliary length is an important and dynamic process and is intimately related to ciliary signaling (28). Cilium extension, which occurs from the basal body, and maintenance of length requires intraflagellar transport (IFT). IFT is a complex process reliant upon molecular motors moving along the microtubules of the ciliary axoneme (29). Defects in ciliary length are associated with severe developmental diseases, including JBTS. Indeed, it is intriguing to note that several renal cystic diseases have been associated with long cilia. Kidney tubules and cultured renal tubular cells from Bbs4-null mice displayed elongated cilia with an intact microtubular structure following prolonged culture (30) whilst *jck* mice (*Nek8* mutation) also revealed long cilia (31,32). Kidney tissues from a foetus affected with *MKS3* mutations displayed longer cilia than control tissue, as measured by alpha-acetylated tubulin staining (33). Cells depleted of KIF7 (JBTSF12) and KIAA0556 (JBTS26) also have extended axonemes (34,35). The KIF7 deficient cilia were both long and had irregular bends along their length and cilia from fibroblasts derived from ovine kidney with mutations in *TMEM67* (JBTS6) have abnormally long cilia, with bulbous defects along the axonemes and sharp kinks (36).

It is clear from these and other reported models of JBTS that alterations of cilium morphology and/or length can have dramatic functional implications. Specifically, elongated cilia take longer to be dismantled, which can influence the timing of the re-entry of the cell into cell cycle (37).

We show that purmorphamine is able to rescue the morphological defects of a JBTS patient’s primary cilia. These data, encouragingly, indicate that ciliopathies are treatable even in hURECs derived from patients at an advanced stage of kidney disease. However, important caveats include the fact that cilium length is not an established biomarker of disease and that purmorphamine treatment has not yet been directly tested using *in vivo* models of *Cep290* knockdown. It also has not been definitively shown that hURECs represent the most relevant cell type to study CEP290-related kidney disease, although these cells are a highly accessible model, and are mostly collecting duct in origin, expressing aquaporin-2 (38,39). It is important to note that the typical kidney disease progression in JBTS patients allows a therapeutic window for intervention with targeted therapies. We show that whilst purmorphamine is a known Hh agonist, treatment of hURECs with this compound result in no changes to canonical Hh target genes but instead produce changes in cell cycle and a reduction in CDK5 levels, suggesting convergence of Hh and CDK pathways. We demonstrate that treatment with either siRNA directed towards CDK5 or roscovitine, a potent inhibitor of CDK5, restores cilia length to JBTS hURECs. This finding seen using JBTS patient kidney cells, is similar to that of the burgeoning number of mouse models that show a positive response to CDK inhibition. Renal cystic kidney disease in *jck* mice, which also exhibit long cilia, can be rescued by CDK inhibition with roscovitine and its more potent and selective analogue S-CR8 (40) as can the cystic phenotype in *cpk* (Cystin) mice (21). Indeed, *Pkd1* conditional knock-out mice also responded to treatment with roscovitine and S-CR8. With the cautions of the interpretation of hURECs and the use of cilia length as a biomarker of cystic kidney disease noted, our data suggests that *CEP290* defects in renal tubular epithelium may be responsive to drug treatments. The morphological rescue in renal cells from patient JBTS II: 2 allows some optimism in targeting and rescuing the renal lesions in CEP290 deficient renal epithelial cells.

It is intriguing to note the similarity between the cilia phenotype we describe for patient-derived renal epithelial cells carrying *CEP290* mutation and the *jck* mouse, caused by mutation of *Nek8*. The increased tortuosity seen under SEM in both cases is remarkably similar and suggests that the ciliary tip lacks a proper tubulin structure in both cases. To date these unrelated genes have not been mechanistically linked, in fact they show quite different localizations with Cep290 protein found in basal bodies and Nek8 protein located along the length of the cilium (41). In fact, Nek8 seems to be localised to a subciliary domain known as the inversin compartment (42). However, the positive response of both *Cep290* and *Nek8* mutant cells (and other ciliopathy models as described above) to CDK inhibitors suggests a common aetiology. Furthermore, our data indicate that Hh agonism with purmorphamine, previously shown to restore cellular phenotype in the case of *Cep290* mutation, may be acting, at least in part, through CDK inhibition. This is underscored by the recent demonstration that a reduction in ciliary length, brought about by CDK inhibitors, attenuates cystic kidney disease in murine nephronophthisis (43) and that our data, obtained from patient-derived renal epithelial cells, indicates that patient cells respond in the same way.

In conclusion, culturing of hURECs from JBTS patients with a cerebello-retinal-renal phenotype with *CEP290* mutations has revealed an elongated cilia phenotype which is rescuable by both purmorphamine, siRNA directed towards CDK5 and the CDK inhibitor roscovitine. We show for the first time that purmorphamine and CDK inhibition treatments have similar phenotypical effects on patient-derived primary kidney cells that seem to reflect a convergence of pathways. This helps to focus the possibilities for therapeutic manipulation of renal ciliopathies.

## Materials and Methods

### Clinical and DNA sequencing

All patients consented to this study. Ethical approval was obtained from the National Research Ethics Service (NRES) Committee North East (14/NE/1076). Following informed and written consent, urine and blood samples were obtained from an affected 4 year-old boy with clinical JBTS with a retinal, renal, and cerebellar phenotype, a 12 year old boy with isolated LCA and his unaffected sibling and healthy gender and age-matched controls. Ethical approval was obtained from the National Research Ethics Service (NRES) Committee North East (14/NE/1076). All methods were performed in accordance with the relevant ethical guidelines and regulations.

Sequencing of ciliopathy genes was performed by applying exon-enriched NGS targeting up to 1, 221 genes associated with cilia including all known genes associated with ciliopathies (“ciliome sequencing”) (44–46) and consecutive barcoded NGS on a SoliD5500 (Life tech) or HiSeq 2500 (Illumina) platform. Confirmation of mutations and segregation analysis was performed on other family members following informed consent using Sanger sequencing, using exon specific primers.

### hUREC isolation, FACS analysis and culture

Human urine-derived renal epithelial cells (hURECs) were isolated from urine collected from patient II: 2 before his onset of ESRD, and healthy age-matched donors and cultured as previously described (2,39). To quantify cell cycle phase distribution, WT and JBST II: 2 cells were fixed in 70% ethanol and treated with RNAse and stained with propidium iodide. The cells were measured using a 5-laser BD LSRII system, measuring PI emission from a 561 nm yellow/green laser in the 610/20 filter. Data was analyzed using BD FACSDiva software.

### Real-time PCR

RNA was extracted using Trizol (Thermo Fisher Scientific) according to the manufacturer’s instructions, and quantified using a NanoDrop 2000 Spectrophotometer (Thermo Fisher Scientific). 1ug of RNA was reverse-transcribed using an Oligo-dT primer and SuperScript III Reverse Transcriptase (Thermo Fisher Scientific). The resulting cDNA was diluted 10-fold in nuclease-free water. Real-time PCR was carried out in a 10ul reaction volume containing the following: 0.5ul of each primer (10uM stock) or 0.5ul PrimeTime^®^ qPCR probe-based assay (IDT) with 0.5ul water, 4ul of cDNA and 5ul of either 2× SYBR Green PCR Master Mix (Applied Biosystems) or 2× PrimeTime^®^ Gene Expression Master Mix (IDT). The PCR was run using a QuantStudio™ 7 Flex Real-Time PCR System (Applied Biosystems) using the following settings: 95 °C for 20 s, followed by 40 cycles of 95 °C for 1 s 60 °C for 20 s. The PrimeTime^®^ assays used were as follows: GAPDH - Hs.PT.39a.22214836, HPRT1-Hs.PT.58.20881146, CEP290 -Hs.PT.58.3248011 and Hs.PT.58.2324908. Primer sequences are available on request.

### Western blotting

48 h after plating or transfection, renal epithelial cells were lysed and proteins were extracted with a 4 M urea, 125 mM Tris pH 6.8, 4% SDS, 10% glycerol, 5% β-mercaptoethanol and 0.02% bromophenol blue solution. Protein samples were heated to 95 °C for 5 min, spun at 16 000 rcf for 5 min and resolved by SDS-PAGE. Proteins were transferred to a nitrocellulose membrane. The membranes were blocked in TBST (Tris-buffered saline, 0.1% Tween-20) containing 5% low fat milk for 1 h then incubated with the following primary antibodies in block overnight at 4 °C [rabbit anti-Cep290, Abcam (ab85728) 1:1000; rabbit anti-Cdk5, New England Biolabs (14145), 1:100; rabbit anti-GAPDH, Cell Signaling technology, 1:5000]. After washing in TBST, membranes were incubated with fluorescently labelled secondary antibodies (LI-COR) for 90 min at room temperature, washed again in TBST and visualized with an Odyssey CLx^®^ imaging system (LI-COR). Quantification was performed with Image studio software (LI-COR).

### Scanning electron microscopy imaging

For SEM, samples were fixed overnight in 2% glutaraldehyde in 0.1 M Sorenson’s phosphate buffer, dehydrated through a graded series of ethanol and critical-point dried (Baltec dryer). They were coated with 10 nm of gold (Polaron coating unit) and viewed on a Tescan Vega LMU SEM operated at 8–10 kV. Images were captured and measured in a blinded fashion.

### Immunofluorescence microscopy imaging

hURECs were fixed in 100% methanol for 10 min. Axoneme of cilia was labelled with monoclonal mouse anti-alpha-acetylated tubulin primary antibody (Sigma, T6793, 1:1000). Ciliary membrane was labelled with rabbit anti-ARL13B (Proteintech, 17711-1-AP). Commercial antibodies were used for detection of Cep162 (Atlas antibodies, rabbit polyclonal, HPA030170), pericentrin (Abcam, rabbit polyclonal, ab4448) and IFT88 (Proteintech, rabbit polyclonal, 13967-1-AP). Fluorescent secondary antibodies used were donkey anti-mouse Alexa Fluor 594 (Thermo Fisher Scientific, 1: 200) and donkey anti-rabbit FITC (Thermo Fisher Scientific, 1: 200). Images and *z*-stacks were captured in a blinded fashion, using the Carl Zeiss Axio Imager and Nikon (A1) confocal inverted microscope.

### siRNA knockdown experiments

hURECs (0.05 × 10^6^) were transfected with 5 pmol Negative Control siRNA (AM4611, ThermoFisher), siRNA targeting human *CDK5* (AM51331, ThermoFisher) or siRNA targeting human *CEP290* (AM16708, ThermoFisher), using Lipofectamine RNAiMAX (ThermoFisher, 13778150) under manufacturer’s instructions.

### Purmorphamine and roscovitine treatments

hURECs were treated with 20 μM Purmorphamine (Merck-Millipore)/0.2% DMSO (Sigma-Aldrich) treatment for 24 h before analysis. hURECs were treated with 10 μM roscovitine (Sigma-Aldrich) for 24 h before fixing and imaging as above. 0.2% DMSO alone was used as a negative control.

## Supplementary Material

Supplementary Material is available at *HMG* online.

## Supplementary Material

Supplementary Figures and Tables
