# Supplementary material for: A human patient-derived cellular model of Joubert syndrome reveals ciliary defects which can be rescued with targeted therapies
Source: Hum Mol Genet. 2017 Sep 11;26(23):4657–67. doi: 10.1093/hmg/ddx347 (PMC5886250; doi:10.1093/hmg/ddx347)
Supplement: Supplementary Figures and Tables [file 1_supplementary_material_revised_final_nomarkings_ddx347.pdf]

Supplementary Material

A human patient-derived cellular model of Joubert syndrome reveals ciliary defects which can be rescued with targeted therapies

Shalabh Srivastava, Simon A. Ramsbottom, Elisa Molinari, Sumaya Alkanderi, Andrew Filby, Kathryn White, Charline Henry, Sophie Saunier, Colin G. Miles & John A. Sayer

Table S1. Common variants identified in the two siblings.

Patients	Genomic position (GRCh37)	Gene	Gene name	RefSeq accession	Nt change c.	AA change p.	Polyphen 2 (score)	Sift (score)	Hom/het	Coverage ref/var (II:2; II:1)	HGMD (gene/variant)	Disease	ExAC (freq)
**Family JBTS**	**Chr12:88496758**	**CEP290**	**Centrosomal protein 290KD**	**NM_025114**	**c.2848dup**	**p.Q950Pfs*6**	-	-	**het**	**(71/81) ; (64/154)**	**Yes/No**	**ciliopathies**	-
	**Chr12:88500452**	**CEP290**	**Centrosomal protein 290KD**	**NM_025114**	**c.2817G>T**	**p.K939N**	**0.771**	**0**	**het**	**(137/125) ; (79/202)**	**Yes/No**	**ciliopathies**	-
	Chr3:132403405	NPHP3	Nephrocystin 3	NM_153240	c.3563A>G	p.K1188R	0.336	0.23	het	(73/99) ; (95/75)	Yes/No	Nephronophthisis	2/121110
	Chr1:220154795	EPRS	Glutamyl-Prolyl-TRNA Synthetase	NM_004446	c.3378G>A	p.M1126I	0.895	0	het	(154/107) ; (95/202)	-	-	-
	Chr2:84775496	DNAH6	Dynein Axonemal Heavy Chain 6	NM_001370	c.1271A>G	p.Y424C	0.976	0.01	het	(109/110) ; (76/163)	Yes/No	Heterotaxy	11/121212
	Chr3:52424969	DNAH1	Dynein Axonemal Heavy Chain 1	NM_015512	c.9640G>A	p.D3214N	0.999	0	het	(23/32) ; (14/30)	Yes/No	Primary ciliary dyskinesia	1/22540
	Chr7:111617266	DOCK4	Dedicator Of Cytokinesis 4	NM_014705	c.622A>G	p.S208G	0.599	0.09	het	(191/177) ; (133/130)	-	-	-
	Chr12:112694200	HECTD4	HECT Domain E3 Ubiquitin Protein Ligase 4	NM_001109662	c.2819C>T	p.T940M	0.293	0	het	(132/121) ; (106/190)	-	-	-

Genes written in bold letters indicate likely disease-causative variants. Columns from left to right: position of the variant in GRCh37 (genomic position), chromosome (Chr), name of the gene in which the variant is detected (Gene), RefSeq accession, nucleotide (Nt) and amino acid (AA) change, Polyphen 2 (PP2) and SIFT Score (red : predict

disease causing, orange: intermediate, green: predict benign), homozygotes (hom) or heterozygotes (het) variants, number of reads whereby position is covered for reference allele (Ref) and variant allele (var), genes and variants reported in Biobase/HGMD and associated disease, allelic frequency in Exome Aggregation Consortium (ExAC).

Table S2. Variants identified only in individual JBTS II:1

Patient	Genomic position (GRCh37)	Gene	Gene name	RefSeq accession	Nt change c.	AA change p.	Polyphen2 (score)	Sift (score)	Hom/het	Coverage ref/var (II:1)	HGMD (gene/variant)	Disease	ExAC (freq)
JBTS II:1	Chr6:38976675	DNAH8	Dynein Axonemal Heavy Chain 8	NM_001206927	c.13300C>T	p.R4434W	1	0	het	(91/110) (54%)	Yes/No	Primary ciliary dyskinesia	35/121288
	Chr2:108455304	RGPD4	RANBP2-Like And GRIP Domain Containing 4	NM_182588	c.289G>C	p.V97L	0.952	0	het	(20/19) (48%)	-	-	-
	Chr2:109379793	RANBP2	RAN binding protein 2	NM_006267	c.2798T>C	p.M933T	0.321	0	het	(103/91) (46%)	Yes/No	Acute necrotizing encephalopathy	1/120748
	Chr14:23511928	PSMB11	Proteasome Subunit Beta 11	NM_001099780	c.494G>A	p.S165N	0.452	0.13	het	(12/10) (45%)	-	-	22/119892
	Chr15:91565406	VPS33B	Vacuolar protein sorting 13B	NM_018668	c.74A>G	p.Q25R	0.006	0.43	het	(36/38) (51%)	Yes/No	Arthrogryposis-renal dysfunction-cholestasis syndrome	0/23306
	Chr3:51378717	DOCK3	Dedicator of cyto-kinesis 3	NM_004947	c.3816C>G	p.D1272E	0.005	0.3	het	(6/8) (57%)	Yes/No	muscle hypotonia, ataxia, and intellectual disability	47/110498

Genes written in bold letters indicate likely disease-causative variants. Columns from left to right: position of the variant in GRCh37 (genomic position), chromosome (Chr), name of the gene in which the variant is detected (Gene), RefSeq accession, nucleotide (Nt) and amino acid (AA) change, Polyphen 2 (PP2) and SIFT Score (red : predict disease causing, orange: intermediate, green: predict benign), homozygotes (hom) or heterozygotes (het) variants, number of reads whereby position is covered for reference allele (Ref) and variant allele (var), genes and variants reported in Biobase/HGMD and associated disease, allelic frequency in Exome Aggregation Consortium (ExAC).

Table S3. Variants identified only in individual JBTS II:2

Patient	Genomic position (GRCh37)	Gene	Gene name	RefSeq accession	Nt change c.	AA change p.	Polyphen2 (score)	Sift (score)	Hom/het	Coverage ref/var (II:2)	HGMD (gene /variant)	Disease	ExAC (freq)
**JBTS II:2**	Chr4:74124072	ANKRD17	Ankyrin Repeat Domain 17	NM_032217	c.303_314 del	p.G107_G110 del	-	-	het	(49/41) (45%)	-	-	2/120728
	Chr3:48019391	MAP4	Microtubule-associated protein 4	NM_002375	c.256A>G	p.N86D	0.703	0.12	het	(56/69) (55%)	Yes/No	Seckel syndrome: growth retardation and normocephaly	1/121400
	Chr15:86262432	AKAP13	A kinase (prka) anchor protein 13	NM_006738	c.6139G>A	p.D2047N	0.998	0	het	(153/152) (49%)	-	-	51/121376
	Chr16:722293	RHOT2	Ras Homolog Family Member T2	NM_138769	c.1235C>T	p.T412M	0.998	0.01	het	(93/98) (51%)	-	-	89/119150
	Chr16:19552027	CCP110	Centriolar Coiled-Coil Protein 110	NM_001199022	c.1967G>A	p.R656Q	0.928	0.08	het	(212/191) (47%)	-	-	81/119426
	Chr17:72741040	RAB37		NM_001163989	c.476G>A	p.R159H	0.871	0.01	het	(132/177) (57%)	-	-	33/121298
	Chr6:38941567	DNAH8	Dynein Axonemal Heavy Chain 8	NM_001206927	c.12656G>A	p.R4219Q	0.001	0.05	het	(64/58) (47%)	Yes/No	Primary ciliary dyskinesia	20/120348
	Chr7:157202588	DNAJB6	DnaJ homologue, subfamily B, member 6	NM_058246	c.791G>A	p.R264Q	0.059	0.69	het	(32/14) (30%)	Yes/No	Myopathy	-
	Chr8:124141323	TBC1D31	TBC1 Domain Family Member 31	NM_145647	c.2135T>C	p.M712T	0.050	0.01	het	(135/115) (46%)	-	-	3/120990
	Chr9:124073114	GSN	Gelsolin	NM_000177	c.657C>G	p.D219E	0.875	0	het	(87/65) (42%)	Yes/No	Familial Amyloidosis of Finnish type, nephrotic syndrome	1/120514
	Chr15:42158038	SPTBN5	Spectrin, beta, non-erythrocytic 5	NM_016642	c.6886C>T	p.R2296W	0.019	0.01	het	(14/5) (26%)	Yes/No	sacral agenesis	2/24600
	Chr22:50969172	ODF3B	Outer Dense Fiber Of Sperm Tails 3B	NM_001014440	c.650A>C	p.K217T	0.142	0.03	het	(62/48) (43%)	-	-	18/98930

Genes written in bold letters indicate likely disease-causative variants. Columns from left to right: position of the variant in GRCh37 (genomic position), chromosome (Chr), name of the gene in which the variant is detected (Gene), RefSeq accession, nucleotide (Nt) and amino acid (AA) change, Polyphen 2 (PP2) and SIFT Score (red : predict disease causing, orange: intermediate, green: predict benign), homozygotes (hom) or heterozygotes (het) variants, number of reads whereby position is covered for reference allele (Ref) and variant allele (var), genes and variants reported in Biobase/HGMD and associated disease, allelic frequency in Exome Aggregation Consortium (ExAC).

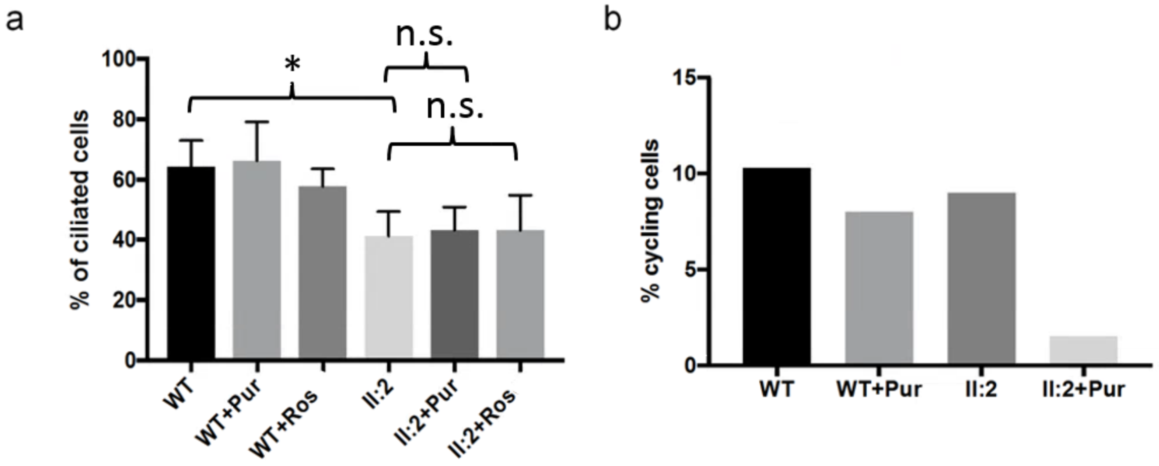

Figure S1: Ciliation rates and FACS analysis of wild type and patient hURECs in response to treatments

(a) Percentage rates of ciliation in serum starved wild type (WT) and JBTS patient II:2 in control conditions and following treatment with Purmorphamine (Pur) and Roscovitine (Ros). There is a significant difference in percentage ciliated cells between WT and II:2 (* $p<0.001$, Unpaired Student's t-test). Treatment of II:2 with Pur or Ros did not change percentage ciliation rates (n.s. not significant).
(b) FACS analysis, using propidium iodide for DNA staining, of wild type (WT) and II:2 hURECs before and after treatment with Purmorphamine (Pur). II:2 cells are sensitive to Pur treatment with a reduction in percentage cycling cells.

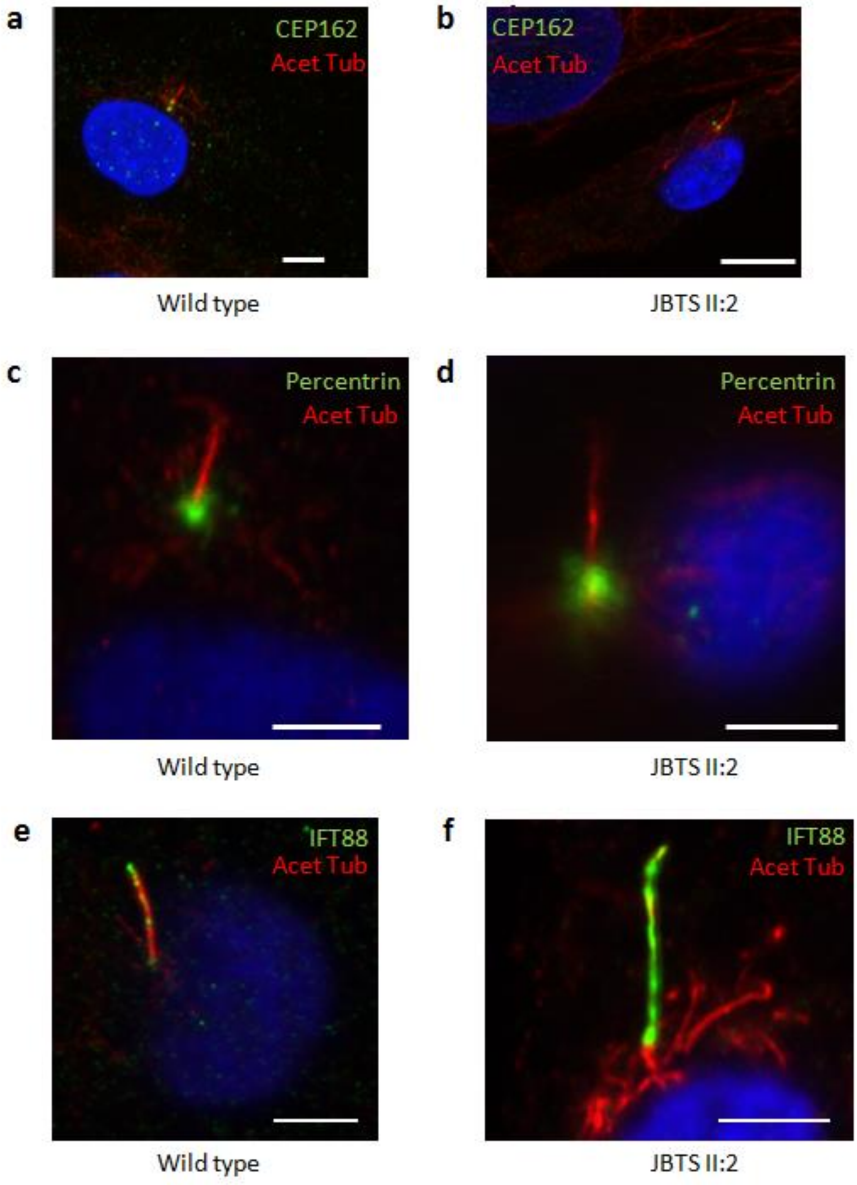

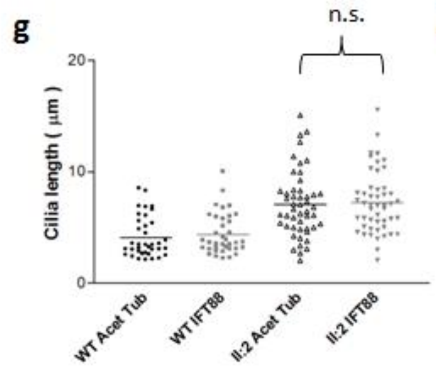

h

Median cilia lengths (μm)

	Wild Type	JBTS II:2
S.E.M.	4.88	7.665
Acet Tub	3.40	4.77
ARL13B	3.85	6.80
IFT88	3.63	5.96

Figure S2: Localisation of transition zone, centrosomal and ciliary proteins in wild type and patient JBTS II:2 hURECs.

Immunofluorescence images showing
(a & b) Wild type and patient JBTS II:2 hURECs with maintained localisation of CEP162 (green), a transition zone protein.

(c & d) Wild type and patient JBTS II:2 hURECs with maintained localisation of pericentrin (green), a centrosomal associated protein.
(e & f) Wild type and patient JBTS II:2 hURECs with localisation of IFT88 (green), an axonemal protein. Cilia are localised with alpha-acetylated tubulin (red). Scale bar 5 µm.
(g) Quantification of ciliary length measured by immunofluorescence imaging using antibodies towards alpha-acetylated tubulin (Acet Tub) and IFT88. There is no significant (n.s. Paired Student's t-test) mismatch in ciliary length between these ciliary axonemal proteins. n numbers: WT cilia = 37, II:2 cilia = 49.
(h) Table showing median cilia length of wild type and JBTS II:2 hURECs when measured by scanning electron microscopy (SEM), and immunofluorescence microscopy using antibodies directed towards alpha-acetylated tubulin (Acet Tub), ARL13B and IFT88.

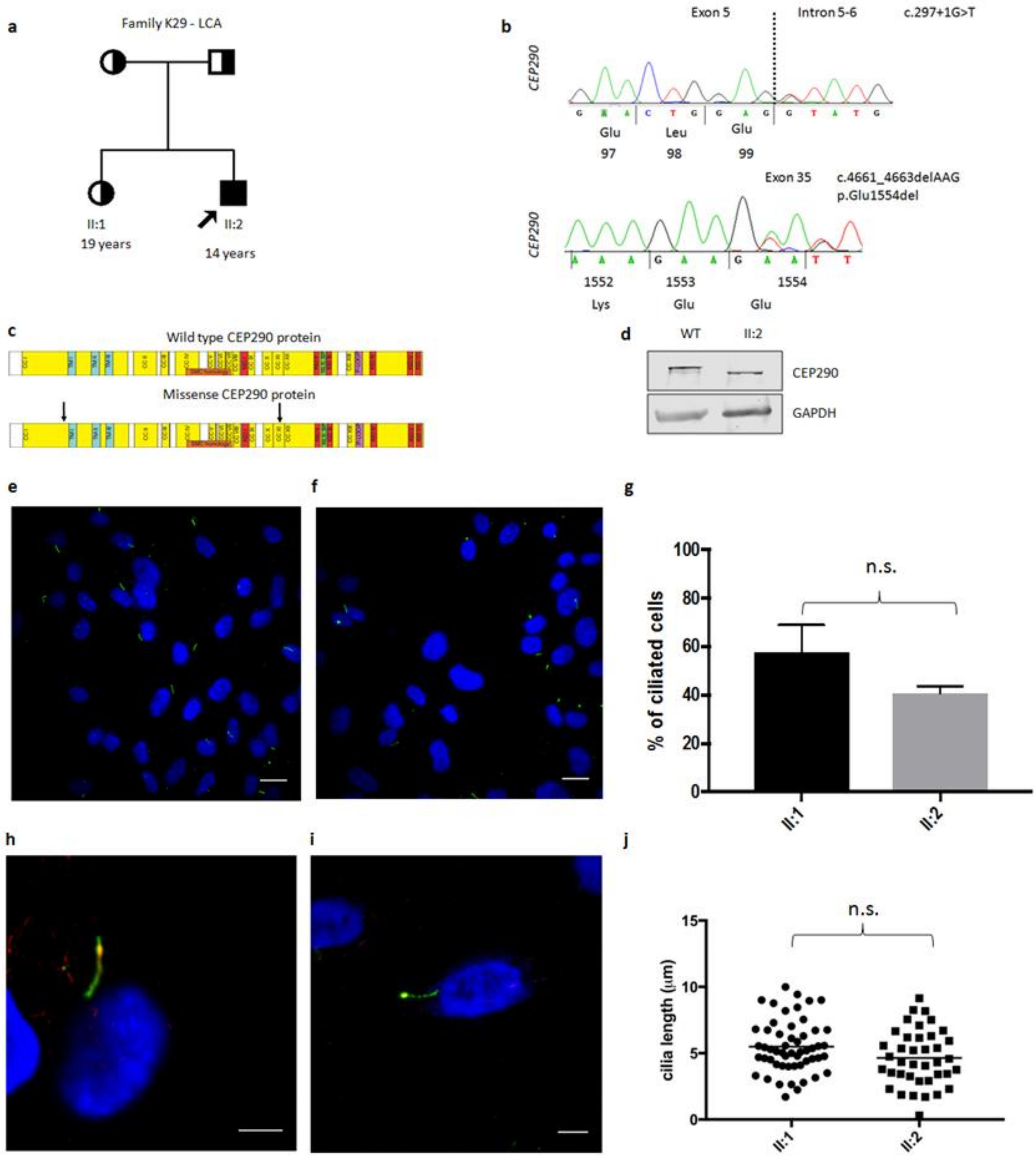

Figure S3: Pedigree, genetic, biochemical and ciliary phenotype data of family LCA

(a) Pedigree diagram showing one affected male with isolated Leber congenital amaurosis (normal renal function and normal brain development (squares, males; circles females)).
(b) Sequence chromatograms from II:2 showing compound heterozygous changes in *CEP290* c.297+1G>T (predicted to affect splice donor site) and c.4661_4663delAAG; p.Q1554del.
(c) Domain structure of CEP290 protein (2479 amino acids) with predicted coiled coil domains (CC) numbered and shown in yellow; tropomyosin homology domain (TM), RepA/Rep^{+} protein KID (KID); bipartite nuclear localization signal (NLS_BP); ATP/GTP-binding site motif A (P-loop). The extent of homology with SMC proteins is indicated by an orange bar. The predicted defects of CEP290 at amino acid positions 99 and 1554 are shown.
(d) Western blot (cropped image) showing normal expression of full-length wild type CEP290 protein from hURECs derived from II:2 using a C-terminal directed CEP290 antibody.
(e) Low power immunofluorescence images (ARL13B-green; DAPI-blue) of hURECs from unaffected sibling II:1 (scale bar = 20 μm).

(f) Low power immunofluorescence images (ARL13B-green; DAPI-blue) of hURECs from affected sibling II:2 (scale bar = 20 µm).
(g) Percentage ciliated hURECs (3 biological replicates) in unaffected II:1 (58%, n= 107) and affected LCA II:2 sibling (41%, n=61), n.s. not significant, Unpaired Student's t-test.
(h) High power immunofluorescence images (ARL13B-green; alpha-acetylated tubulin-red; DAPI-blue) of hURECs from unaffected sibling II:1 (scale bar = 5 µm).
(i) Low power immunofluorescence images (ARL13B-green; DAPI-blue) of hURECs from affected sibling LCA II:2 (scale bar = 5 µm).
(j) Dot plot with means of hUREC cilia length in unaffected sibling (II:1) (n= 54) and affected sibling LCA II:2 (n=39), n.s. not significant, Unpaired Student's t-test.

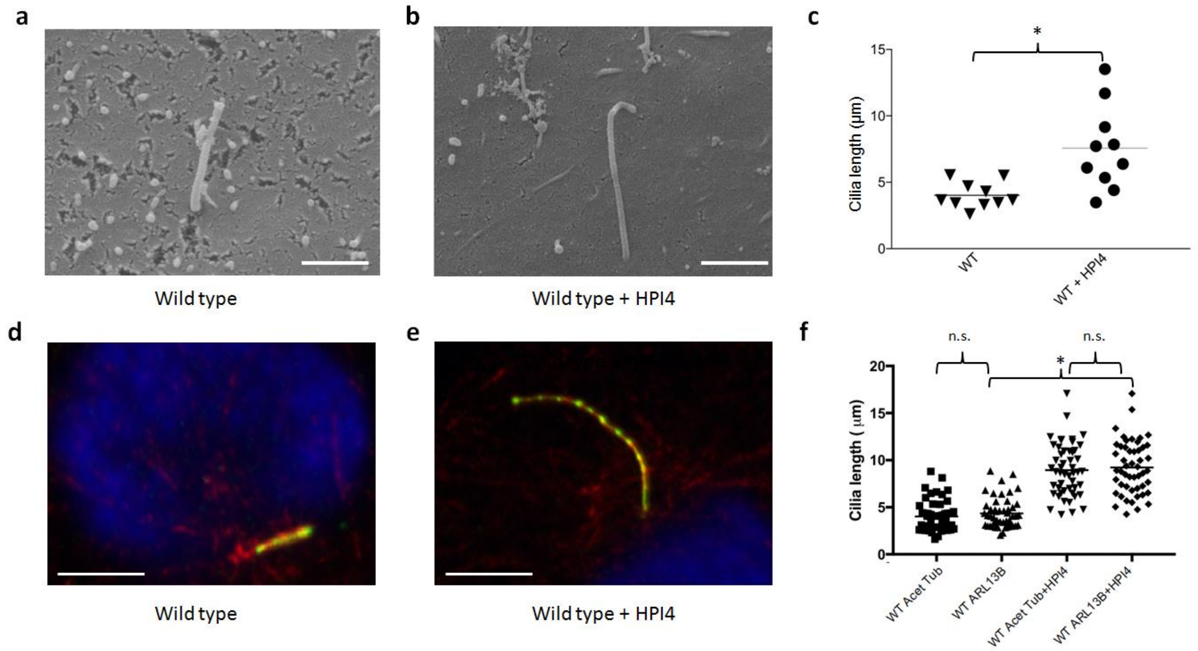

Figure S4: Hh pathway manipulation of wild–type cilia in hURECs

(a) SEM image of cilia from wild type hURECs (Scale bar 2 µm).
(b) SEM images of cilia from wild type hURECs treated with Hh antagonist HPI-4 (Scale bar 2 µm).
(c) Dot plot with means showing quantification of ciliary lengths following EM imaging. * $p<0.001$, Unpaired Student's t-test.
(d) Immunofluorescence imaging (alpha-acetylated tubulin red; ARL13B green) of cilia from wild type hURECs (scale bar 5 µm).
(e) Immunofluorescence imaging (alpha-acetylated tubulin, red; ARL13B, green) of cilia from wild type hURECs treated with HPI-4 (Scale bar 5 µm).
(f) Dot plot with means showing quantification of ciliary lengths following immunofluorescence imaging (Acet Tub, alpha-acetylated tubulin; n.s. not significant, *, $p< 0.001$, Unpaired Student's t-test).

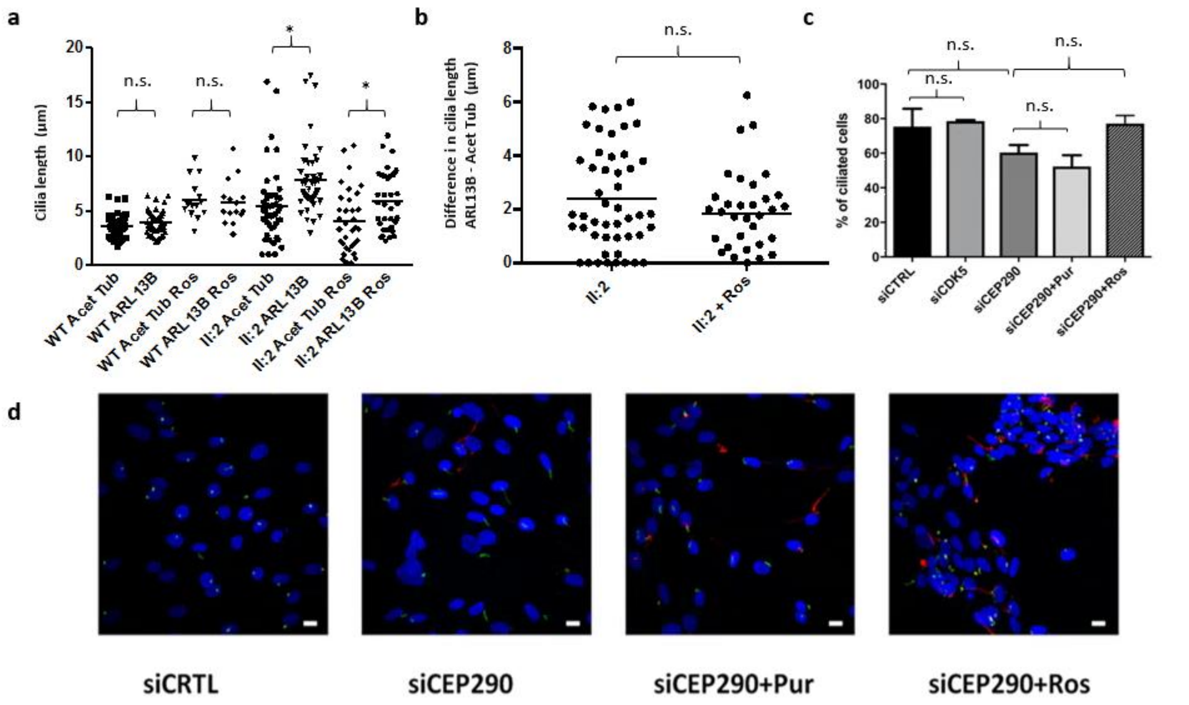

Fig. S5: Response to treatments in II:2 hURECS and CEP290 depleted wild type hURECS

(a) Dot plot with means to show quantification of cilia length using both alpha-acetylated tubulin (Acet Tub) and ARL13B in wild type (WT, n=48), wild type treated with roscovitine (WT + Ros, n=15), JBTS II:2 hURECs (n=48) and JBTS II:2 treated with roscovitine (Ros, n=34) treatment (n.s. not significant, * p<0.0001, Paired Student's t- test).

(b) Dot plots with means indicated to show the difference in cilia length of each cilia (data from panel a) as determined by the measuring axonemal length under immunofluorescence imaging using antibodies against alpha-acetylated tubulin (Acet Tub) and ARL13B. (n.s, not significant, Unpaired Student's t-test)

(c) Percentage rates of ciliation in serum starved wild type cells treated with control siRNA (siCTRL); siRNA CDK5, (siCDK5); siRNA CEP290 (siCEP290); siRNA CEP290 + Purmorphamine (siCEP290+Pur) and siRNA CEP290 + Roscovitine (siCEP290+Ros). Experiments were performed in triplicate. The difference in percentage ciliation rates for siCTRL, siDCDK5, siCEP290 and following Pur or Ros treatment was not significant (n.s. ANOVA).

(d) Low power images showing ciliation rates in wild type hURECs treated with control siRNA (siCTRL); siRNA CEP290 (siCEP290); siRNA CEP290 + purmorphamine (siCEP290+Pur) and siRNA CEP290 + roscovitine (siCEP290+Ros). Scale bar 10 µm.
